# Sex-specific clinicopathological significance of novel (Frizzled-7) and established (MGMT, IDH1) biomarkers in glioblastoma

**DOI:** 10.18632/oncotarget.10465

**Published:** 2016-07-07

**Authors:** Salveena Schiffgens, Ludwig Wilkens, Alba A. Brandes, Tatiana Meier, Enrico Franceschi, Mario Ermani, Christian Hartmann, Ibrahim Erol Sandalcioglu, Claudia A. Dumitru

**Affiliations:** ^1^ Department of Neurosurgery, Nordstadt Hospital Hannover, Hannover, Germany; ^2^ Department of Pathology, Nordstadt Hospital Hannover, Hannover, Germany; ^3^ Department of Medical Oncology, Bellaria Hospital, AUSL-IRCCS Institute of Neurological Sciences, Bologna, Italy; ^4^ Department of Neurosciences, Statistics and Informatics Unit, University Hospital Padova, Padua, Italy; ^5^ Department of Neuropathology, Institute of Pathology, Hannover Medical School, Hannover, Germany

**Keywords:** Frizzled-7, MGMT, IDH1, sex-specific biomarkers, glioblastoma

## Abstract

**Background:**

The Wnt receptor Frizzled-7 (FZD7) promotes tumor progression and can be currently targeted by monoclonal antibody therapy. Here, we determined the prognostic value of FZD7 for the overall survival of glioblastoma (GBM) patients, both as individual marker and taken in combination with the previously-described markers MGMT and IDH1. Additionally, we tested whether these markers (alone or in combination) exhibited sex-specific differences.

**Results:**

High levels of FZD7 (FZD7^high^) associated with shorter survival in GBM patients; however, FZD7^high^ was a significant predictor of poor survival only in male patients. Mutation of IDH1 significantly associated with longer survival in male but not female patients. Methylated MGMT promoter significantly associated with longer survival only in female patients. Combination of FZD7 with MGMT enhanced the prognostic accuracy and abrogated the sex differences observed upon single marker analysis. Combination of FZD7 with IDH1 was a significant predictor of survival in male GBM patients only.

**Materials and Methods:**

Three independent cohorts of patients with primary GBM (n=120, n=108 and n=105, respectively) were included in this study. FZD7 and IDH1 were assessed by immunohistochemistry in tissue microarrays. MGMT promoter methylation was determined by methylation-specific polymerase chain reaction. Survival analysis was performed by Kaplan-Meier estimate, log-rank test and Cox proportional hazard regression.

**Conclusions:**

Our study identifies novel individual and combination markers with prognostic and, possibly, therapeutic relevance in GBM. Furthermore, our findings substantiate the importance of sexual dimorphism in this type of cancer.

## INTRODUCTION

Glioblastoma (GBM) is the most common and aggressive malignant brain tumor in adults with an incidence of 0.59-3.69 cases per 100,000 person life-years [1]. The vast majority of GBM develop *de novo* (primary GBM); however, GBM can also evolve from lower grade gliomas (secondary GBM). Primary GBM occur more commonly in male patients whereas the reverse is the case for secondary GBM [2]. The mean age of primary and secondary GBM patients is 62 and 45 years, respectively [2]. Currently, the standard of care treatment for GBM consists of maximum safe surgical resection followed by radiotherapy plus concomitant and adjuvant chemotherapy with temozolomide [3]. Despite this aggressive therapeutic regimen, GBM patients have a very poor prognosis, with only 0.05-4.7% of patients surviving 5 years past initial diagnosis [1]. Although a large body of research addressed potential molecular markers and targets in GBM, this disease remains incurable at present. Thus, there is still an urgent need to identify novel clinicopathological factors with a superior accuracy regarding diagnosis, prognosis and therapeutic prediction in GBM patients.

Frizzled-7 (FZD7) is a member of the Frizzled family of transmembrane proteins that serve as receptors for the Wnt ligands [4]. Like other members of this family, FZD7 can bind to more than one ligand thereby activating the canonical and/or the non-canonical Wnt signaling pathways. Subsequently, a plethora of transcription factors are switched on, which are essential for modulation of important cellular functions such as proliferation, migration, polarity or differentiation [4]. Accumulating evidence indicates that aberrant activation of the FZD7/Wnt pathway promotes carcinogenesis and tumor progression. Specifically, FZD7 is overexpressed in various solid cancers (reviewed in [4]) and high levels of FZD7 associate with shorter survival of colorectal and gastric cancer patients [5, 6]. Recently, inhibition of FZD7 by the therapeutic monoclonal antibody OMP-18R5 was shown to decrease tumor growth and tumorigenicity of several human xenograft tumors [7].

The role of FZD7 in GBM is currently unknown. The objective of this study was to determine the expression and prognostic value of FZD7 in patients with primary GBM. Furthermore, we sought to identify novel strategies for a more accurate prognosis in these patients by analyzing FZD7 in combination with the previously-described markers MGMT (O-6-methylguanine-DNA methyltransferase) promoter methylation [8] and IDH1 (isocitrate dehydrogenase 1) mutation status [9]. As several studies described the sexually-dimorphic expression of cancer biomarkers including MGMT [10–12], we additionally tested whether our markers exhibited sex-specific differences in relationship to the overall survival of GBM patients.

## RESULTS

**Figure 1:**
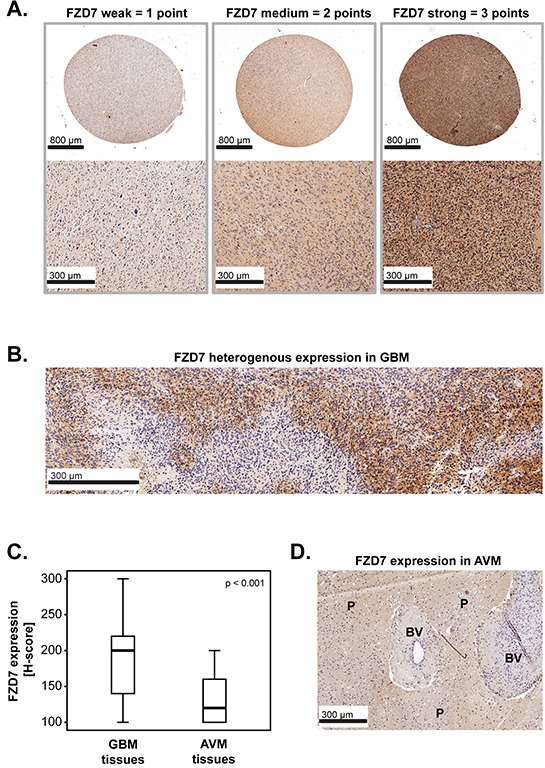
FZD7 in primary GBM: expression and scoring system **A-B.** Representative micrographs showing (A) weak, medium or strong expression of FZD7 and (B) heterogenous expression of FZD7 in GBM tissues. Scale bars are indicated in the lower-left corner of each panel. **C.** FZD7 expression in GBM tissues (n=222) versus non-cancerous brain tissues (brain parenchyma from patients with arteriovenous malformations-AVM; n=52). The medians are shown as black lines and the percentiles (25th and 75th) as vertical boxes with error bars. Statistical analysis was performed with the chi-square test and the p-value is indicated in the upper right corner of the plot. **D.** Representative micrograph showing FZD7 expression in AVM tissues. The scale bar is indicated in the lower-left corner of the panel. BV= blood vessels; P=brain parenchyma.

FZD7 scoring (Figure 1A-1B) and comparison of FZD7 expression between GBM and non-cancerous brain tissues (Figure 1C-1D) are described in detail in the Material and Methods section.

### FZD7 and overall survival (OS) of GBM patients

**Figure 2:**
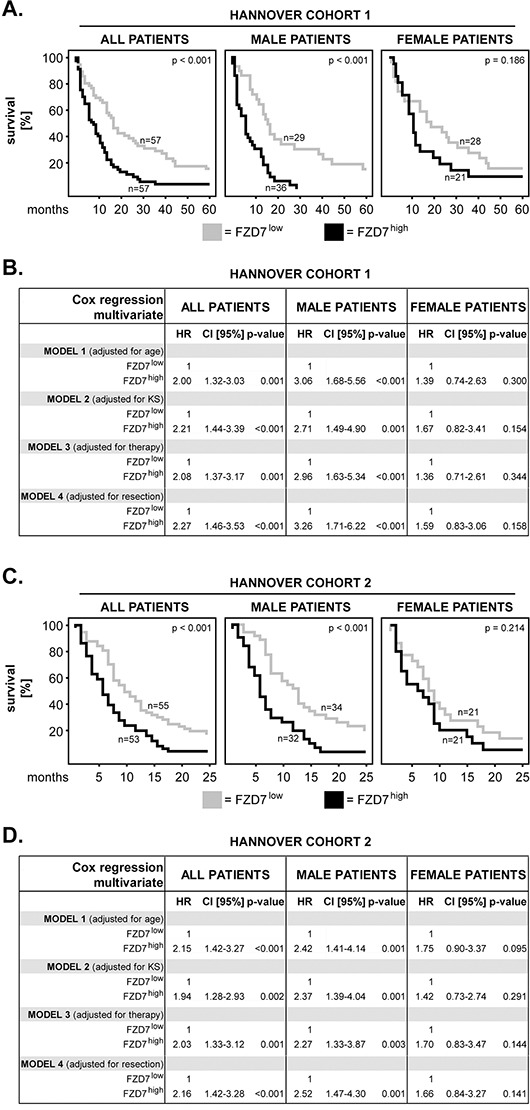
FZD7 in primary GBM: impact on patients’ overall survival and sex differences **A.** Kaplan–Meier 5-year survival curves were plotted for the Hannover Cohort 1 patients with low versus high levels of FZD7. Analysis was performed for the entire cohort of patients (all patients), male and female patients, respectively. The log-rank test was used for statistical testing and the p-values are indicated in the upper-right corner of each plot. The numbers of patients per group are indicated next to each curve. **B.** Multivariate Cox regression analysis models for the Hannover Cohort 1 patients with FZD7^low^ versus FZD7^high^. The models were adjusted for age, Karnofsky scale (KS), therapy and extent of surgical resection, respectively. HR = hazard ratio; 95% CI = 95% confidence interval. **C.** Kaplan–Meier 2-year survival curves were plotted for the Hannover Cohort 2 patients with low versus high levels of FZD7. The statistical testing was performed as in (A). **D.** Multivariate Cox regression analysis models for the Hannover Cohort 2 patients with FZD7^low^ versus FZD7^high^. The statistical testing was performed as in (B).

We first determined whether there might be a relationship between the expression of FZD7 and the overall survival (OS) of GBM patients. Furthermore, we tested whether this association might be sex specific. To this end, FZD7 levels were dichotomized into FZD7^low^ and FZD7^high^ based on the median split method. Survival curves were plotted according to the Kaplan-Meier method and significance was tested with the log-rank test. The results showed that high levels of FZD7 (FZD7^high^) significantly associated with shorter survival in the entire cohort of patients (p<0.001; log rank) and in male patients (p<0.001; log rank); however, this association did not reach statistical significance in female patients (p=0.186; log rank) (Figure 2A). Additionally, multivariate analysis using age, Karnofsky scale (KS), therapy or surgical resection as covariates showed that FZD7^high^ significantly predicted poor survival in the entire cohort and in male patients, but not in female patients (Figure 2B). These findings were confirmed in an independent group of GBM patients (Hannover Cohort 2), where we found that FZD7^high^ significantly associated with shorter survival in the entire cohort of patients (p<0.001; log rank) and in male patients (p<0.001; log rank) but not in female patients (p=0.214; log rank) (Figure 2C). Multivariate analysis of this second GBM cohort additionally confirmed that FZD7^high^ was a significant predictor of poor survival in the entire cohort and in male patients, but not in female patients (Figure 2D).

### MGMT methylation status and OS of GBM patients

**Figure 3:**
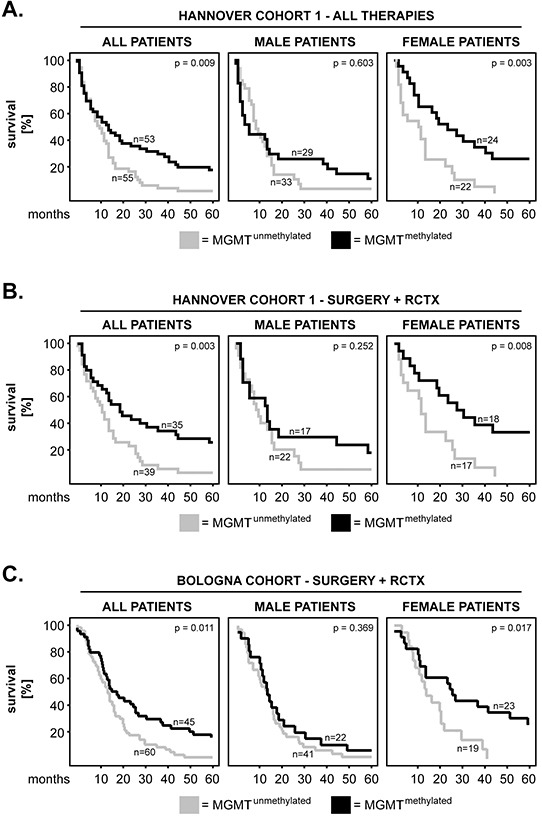
MGMT methylation in primary GBM: impact on patients’ overall survival and sex differences **A-B.** Kaplan–Meier 5-year survival curves were plotted for the Hannover Cohort 1 patients with methylated versus unmethylated MGMT promoter. Analysis was performed for (A) all therapy groups and (B) only the group which received radiochemotherapy (RCTX) in addition to surgery. **C.** Kaplan–Meier 5-year survival curves were plotted for the Bologna patients with methylated versus unmethylated MGMT promoter. All patients in this cohort had received RCTX in addition to surgery. The p-values of the log-rank test are indicated in the upper-right corner of each plot. The numbers of patients per group are indicated next to each curve.

Previous studies showed that the methylation status of MGMT was important for the clinical outcome of GBM patients [8]. We, therefore, investigated the impact of the methylated MGMT form (MGMT^methylated^) versus the unmethylated form (MGMT^unmethylated^) on the survival of our patients. We found that MGMT^methylated^ significantly associated with longer survival in the entire cohort of patients (p=0.009; log rank) and in female patients (p=0.003; log rank) but, interestingly, not in male patients (p=0.603; log rank) (Figure 3A). As the methylation status of MGMT influences the survival of GBM patients mainly upon treatment with alkylating chemotherapeutic agents [13], we further analysed only the patients that received chemotherapy in addition to radiotherapy and surgery (Figure 3B). The results showed that MGMT^methylated^ remained significantly associated with longer survival in the whole group of patients (p=0.003, log rank) and in female patients (p=0.008; log rank), whereas it remained insignificant in male patients (p=0.252; log rank) (Figure 3B). Importantly, these findings were confirmed in an independent GBM patient group (the Bologna cohort), where we found that MGMT^methylated^ was significantly associated with longer survival in the entire cohort (p=0.011, log rank) and in female patients (p=0.017; log rank), but not in male GBM patients (p=0.369; log-rank) (Figure 3C).

### IDH1 mutation status and OS of GBM patients

**Figure 4:**
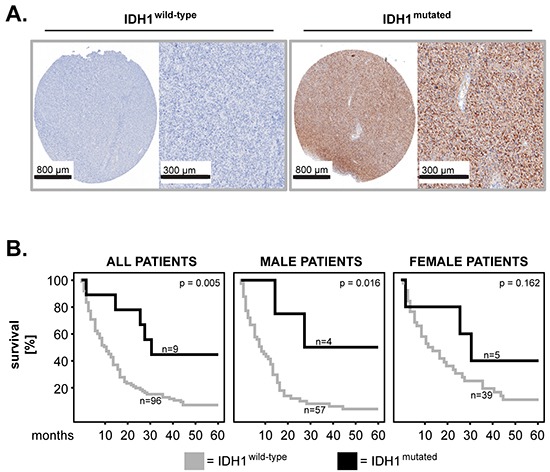
IDH1 mutation in primary GBM: impact on patients’ overall survival and sex differences **A.** Representative micrographs showing expression of wild-type IDH1 (negative staining; left panels) and mutated IDH1 (positive staining; right panels). Scale bars are indicated in the lower-left corner of each panel. **B.** Kaplan–Meier 5-year survival curves were plotted for the Hannover Cohort 1 patients with mutated versus wild-type status of IDH1 and statistical testing was performed with the log-rank test.

Another marker of potential relevance for the outcome of GBM patients is IDH1. Specifically, it was shown that IDH1 mutations associated with prolonged survival of GBM patients [9]. In this study we assessed the R132H mutation of IDH1 by immunohistochemistry. Figure 4A depicts examples of mutated IDH1 (positive staining; IDH1^mutated^) and wild-type IDH1 (negative staining; IDH1^wild-type^), respectively (Figure 4A). Kaplan-Meier analysis showed that IDH1^mutated^ significantly associated with longer survival in the entire cohort of GBM patients (p=0.005; log rank) (Figure 4B). Sex-specific analysis revealed a significant association between the mutation status of IDH1 and survival in male but not female patients (p=0.016 and p=0.162, respectively; log rank) (Figure 4B). The mutation status of IDH1 was additionally assessed in the Hannover Cohort 2 patients; however this cohort did not contain any IDH1^mutated^ specimens (data not shown).

### Combination of FZD7 with MGMT or IDH1 and OS of GBM patients

**Figure 5:**
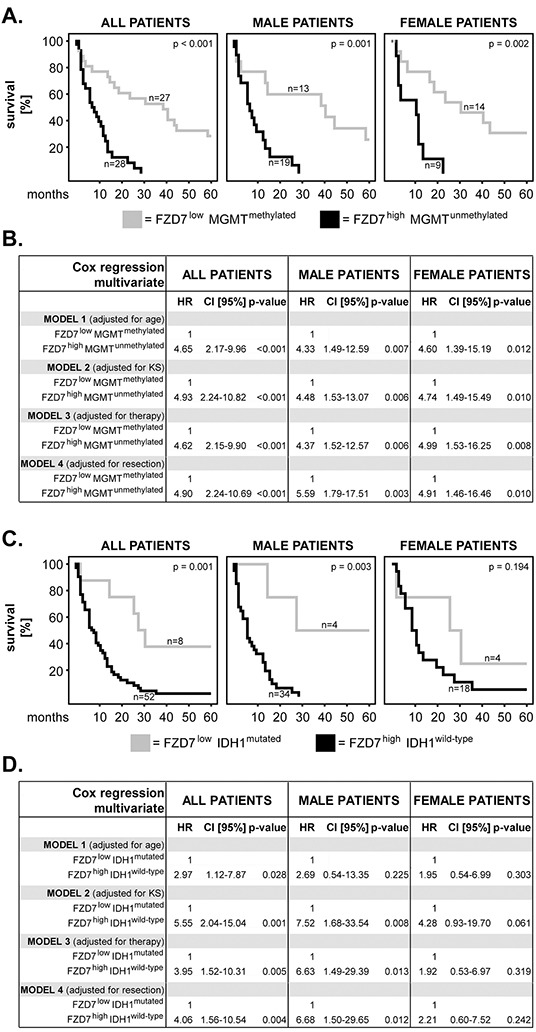
Combination of FZD7 with MGMT or IDH1: impact on patients’ overall survival and sex differences **A.** Kaplan–Meier 5-year survival curves and **B.** multivariate Cox regression analysis models for the Hannover Cohort 1 patients with FZD7^low^MGMT^methylated^ versus FZD7^high^MGMT^unmethylated^ phenotypes. **C.** Kaplan–Meier 5-year survival curves and **D.** multivariate Cox regression analysis models for the Hannover Cohort 1 patients with FZD7^low^IDH1^mutated^ versus FZD7^high^IDH1^wild-type^ phenotypes. (A, C) Statistical testing was performed with the log-rank test and the p-values are indicated in the upper-right corner of each plot. The numbers of patients per group are indicated next to each curve. (B, D) The multivariate models were adjusted for age, Karnofsky scale (KS), therapy and extent of surgical resection. HR = hazard ratio; 95% CI = 95% confidence interval.

In the final set of studies we tested the impact of FZD7 taken in combination with MGMT or with IDH1 on the survival of GBM patients. To this end we combined the phenotypes which, in single marker analysis, associated with the longest and the shortest survival, respectively. Thus, we compared FZD7^low^MGMT^methylated^ with FZD7^high^MGMT^unmethylated^ and FZD7^low^IDH1^mutated^ with FZD7^high^IDH1^wild-type^. We found that the FZD7^high^MGMT^unmethylated^ phenotype significantly associated with shorter survival in the entire cohort of patients (p<0.001; log rank) (Figure 5A). Notably, this association was significant in both male (p=0.001; log rank) and female (p=0.002; log rank) GBM patients (Figure 5A). Furthermore, multivariate analysis showed that the FZD7^high^MGMT^unmethylated^ phenotype significantly predicted poor survival in all groups of patients (Figure 5B).

The FZD7^high^IDH1^wild-type^ phenotype significantly associated with poor survival in the entire cohort and in male patients (p=0.001 and p=0.003, respectively; log rank) but not in female patients (p=0.194; log rank) (Figure 5C). Multivariate analysis showed that the FZD7^high^IDH1^wild-type^ phenotype predicted poor survival in the entire cohort of patients (Figure 5D). In male patients, FZD7^high^IDH1^wild-type^ significantly predicted survival when the models were adjusted for Karnofsky scale, therapy and surgical resection, but lost its significance when the model was adjusted for age (Figure 5D). In female patients, the FZD7^high^IDH1^wild-type^ phenotype did not have any prognostic value (Figure 5D).

## DISCUSSION

This is the first report showing that high expression of FZD7 significantly associates with shorter overall survival of GBM patients and predicts poor survival of these patients. FZD7 might, therefore, serve as an independent prognostic biomarker in primary GBM. Notably, FZD7 can be inhibited by the therapeutic monoclonal antibody Vantictumab (OMP-18R5) which showed potent anti-tumor effects in murine xenograft studies [7] and is currently being tested in clinical trials for various solid cancers. Hence, FZD7 may also represent a novel therapeutic target in GBM patients.

Interestingly, the association between FZD7 expression and patients’ survival displayed sex differences, as high levels of FZD7 significantly associated with shorter survival only in male GBM patients. Sex differences were also observed for the other two markers analysed in this study - MGMT and IDH1. Apart from the higher incidence rate of primary GBM in male patients [1], little is known about the role of sexual dimorphism in GBM. Very recent evidence indicates, however, that sexual dimorphism might significantly modulate the biology of GBM. For instance, astrocytes derived from male NF1^−/−^ DNp53 mice (mesenchymal GBM model) exhibited higher proliferation and colony-forming abilities *ex vivo*, as well as enhanced tumorigenesis *in vivo* compared to astrocytes derived from their female counterparts [14]. Colen and co-workers found that GBM tumors from male patients had increased necrosis than those from female patients [15]. Further transcription factor analysis showed that tumor cell death associated with MYC in female patients and with TP53 activity in male GBM patients [15]. Together with our own studies, these findings suggest that sexual dimorphism is an important factor in the pathophysiology of GBM, which should most likely be taken into consideration in the management of this disease.

Accumulating evidence indicates that combined analysis of multiple markers has superior prognostic value compared to analysis of individual markers [16–19]. Here, we combined FZD7 with MGMT or IDH1, as these markers were shown to associate with the survival of GBM patients in previous [8, 9] and present (Figures 3–4) studies. We found that FZD7/MGMT combination was a strong independent predictor of poor survival in GBM patients, with twice as high hazard ratios compared to FZD7 alone. Notably, this combination of markers also abrogated the sex differences observed upon single marker analysis. Thus, combining FZD7 with MGMT might be a good strategy for an accurate prognosis in primary GBM patients. Combination of FZD7 with IDH1 revealed considerably higher hazard ratios in male patients compared with FZD7 alone, but lost its significance when adjusted for age. This phenomenon might be a consequence of the high prevalence of mutated IDH1 in younger GBM patients [20, 21]. Furthermore, the 95% confidence intervals were very wide (probably because of the scarcity of IDH1 mutated cases) and no IDH1 mutations were found in a second cohort of primary GBM. These findings are in agreement with previous studies showing that IDH1 mutations in primary GBM are rare [22, 23] and, therefore, warrant much larger patient cohorts for validation.

In summary, our study identifies novel individual (FZD7) and combination (FZD7/MGMT) prognostic markers and substantiates the importance of sexual dimorphism in GBM. These findings contribute to a better understanding of GBM pathophysiology and might ultimately foster the development of novel therapeutic strategies against this type of cancer.

## MATERIALS AND METHODS

### Study subjects

Three independent patient cohorts were used in this study: Hannover Cohort 1, Hannover cohort 2 and the Bologna Cohort. All patients had histopatologically-confirmed GBM. These tumors were clinically classified as primary GBM as no lower graded glioma was present in the patients’ medical history. Hannover Cohort 1 included 120 GBM patients with a median age of 64.5 years. The patients were treated at the Department of Neurosurgery, Nordstadt Hospital Hannover between 2000 and 2010. Hannover Cohort 2 included 108 patients with a median age of 68 years. The patients were treated at the Department of Neurosurgery, Nordstadt Hospital Hannover between 2010 and 2014. All studies were approved by the ethics committee of the Medical School Hannover. The data were analysed anonymously and the ethics committee provided a waiver of the need for informed consent. The Bologna Cohort included 105 patients recruited between 2006 and 2010, with a median age of 54.2 years. All studies were approved by the ethics committee of the University Bologna. Patients characteristics including sex, Karnofsky scale (KS), therapy and extent of surgical resection are summarized in Table 1. Hannover Cohort 1 was used in all studies. Hannover Cohort 2 was used in the studies investigating the relationship between FZD7 or IDH1 and the patients’ survival. The Bologna Cohort was used in the studies investigating the relationship between MGMT methylation status and the patients’ survival.

**Table 1:** Patient characteristics

	HANNOVER COHORT 1		HANNOVER COHORT 2		BOLOGNA COHORT	
	Number	%	Number	%	Number	%
all patients	120	100	108	100	105	100
**Sex**						
female	51	42.5	42	38.9	42	40
male	69	57.5	66	61.1	63	60
**Karnofsky scale (KS)**						
10	1	0.8	1	0.9	0	0
20	1	0.8	0	0	0	0
30	1	0.8	2	1.9	0	0
40	3	2.5	11	10.2	0	0
50	13	10.8	18	16.7	0	0
60	15	12.5	31	28.7	2	1.9
70	16	13.3	31	28.7	7	6.7
80	41	34.2	3	2.8	26	24.8
90	21	17.5	10	9.3	56	53.3
100	0	0	1	0.9	14	13.3
n.d.	8	6.7	0	0	0	0
**Therapy**						
surgery	11	9.2	13	12	0	0
CTX	1	0.8	0	0	0	0
surgery + RTX	19	15.8	10	9.3	0	0
surgery + CTX	3	2.5	2	1.9	0	0
surgery + RCTX	82	68.3	76	70.4	105	100
n.d.	4	3.3	7	6.5	0	0
**Surgical resection**						
total	57	47.5	34	31.5	49	46.6
subtotal/biopsy	51	42.5	73	67.6	56	53.3
n.d.	12	10.0	1	0.9	0	0

CTX=chemotherapy; RTX=radiotherapy; RCTX=radiochemotherapy; n.d.=not determinable

Additionally, FZD7 expression was tested on tissues from patients with brain arteriovenous malformations (AVM) (n= 52). These studies were approved by the ethics committee of the Medical School Hannover as well.

### Tissue microarrays (TMA): construction and immunohistochemistry

TMA blocks were built using the Arraymold kit E (Riverton, UT, USA). Briefly, tissue cores derived from vital and solid tumor areas were extracted from formalin-fixed/paraffin-embedded (FFPE) GBM tissues using a 3 mm biopsy punch. The cores were transferred into recipient blocks and cut into 2 μm sections. Prior to staining, the sections were deparaffinized and the antigens were retrieved by heat-induced antigen retrieval (HIER) in citrate buffer pH 6.0 (Thermo Scientific, Freemont, CA, USA). For FZD7 immunohistochemistry, the sections were stained with 2 μg/ml polyclonal rabbit anti-human FZD7 antibodies (Acris Antibodies, Herford, Germany) overnight at 4°C. Secondary and colorimetric reactions were performed using the UltraVision™ LP Detection System according to the manufacturer's instructions (Thermo Scientific). Nuclei were counterstained with Haematoxylin (Carl Roth, Karlsruhe Germany) and the sections were covered with Mountex (Medite, Burgdorf, Germany). For IDH1, samples were stained with 6.6 μg/ml monoclonal mouse anti-human IDH1 R132H (clone H09) antibodies (Dianova, Hamburg, Germany) according to the protocol described in [24]. All stained TMAs were digitalized with an Aperio AT2 high resolution whole-slide scanner and the digital images were viewed with the Aperio ImageScope software (both from Leica Biosystems, Nussloch, Germany). Blinded histological analysis was performed independently by authors S.S., C.A.D., L.W. (senior histopathologist) and C.H (senior neuropathologist).

### FZD7: expression and scoring system

Expression of FZD7 was determined by immunohistochemistry using tissue microarrays (TMAs) from patients with primary GBM. The expression intensity of FZD7 was categorized as weak, medium and strong and was assigned 1, 2 and 3 points, respectively (Figure 1A). As a number of samples exhibited heterogeneous staining (Figure 1B), FZD7 expression was graded using the H-score according to the formula: (1 × *x*)+(2 × *y*)+(3 × *z*), where *x* + *y* + *z* =100% of the tumor area.

Additionally, FZD7 expression was determined in non-cancerous brain tissues (brain parenchyma from AVM patients) using the same scoring system as above. The results demonstrated that the levels of FZD7 were significantly higher in GBM tissues compared to AVM tissues (p<0.001; chi-square) (Figure 1C). A representative example of FZD7 expression intensity in AVM tissues is shown in Figure 1D.

### MGMT promoter methylation analysis

For the Hannover cohort, macrodissected FFPE GBM tissues were deparaffinized with xylene and genomic DNA (gDNA) was isolated using the QIAamp DNA FFPE Tissue kit (Qiagen, Hilden, Germany). The gDNA concentration and purity was assessed using a NanoDrop ND-1000 UV-VIS spectrophotometer (PeqLab, Erlangen, Germany). The isolated gDNA was subjected to bisulfite conversion using the EpiTect Bisulfite Kit (Qiagen) followed by methylation-specific polymerase chain reaction (MSP) using the primer sets established by Esteller et al. [25]. EpiTect bisulfide converted unmethylated and methylated human DNAs (Qiagen) as well as samples without any DNA were included as controls. The PCR products were separated by electrophoresis on 3% agarose gels. Gel imaging and analysis was performed with BioDocAnalyse Sytems (Biometra, Göttingen, Germany). The tumor specimens which showed a visible band of methylated PCR product were considered as *methylated,* whereas the absence of this band indicated *unmethylated* tumor samples.

For the Bologna cohort, the MGMT promoter methylation analysis was performed exactly as described previously [26].

### Statistical analysis

Clinical data were analyzed with the SPSS statistical software version 18.0 (SPSS Inc, Chicago, IL, USA). Differences in FZD7 expression between GBM and AVM tissues were determined using the chi-square test. Survival curves (5-year or 2-year cutoff) were plotted according to the Kaplan–Meier method. Significance was initially tested by univariate analysis using the log-rank test. Multivariate analysis was used to determine the prognostic value of selected variables using Cox's proportional hazard linear regression models adjusted for age, Karnofsky scale (KS), therapy and extent of surgical resection, as these factors are known to influence the prognosis of GBM patients. In all studies, the level of significance was set at p ≤ 0.05.
